# Spatiotemporal variation characteristics and influencing factors of snow density in the High Mountain Asia from 1960 to 2023

**DOI:** 10.1371/journal.pone.0338605

**Published:** 2025-12-16

**Authors:** Zhankun Wang, Dong Cui, Baofu Li, Haijun Liu

**Affiliations:** 1 School of Resources and Environment, Yili Normal University, Yining, China; 2 School of Geography and Tourism, Qufu Normal University, Rizhao, China; Sejong University, KOREA, REPUBLIC OF

## Abstract

Under the context of climate change, significant variations in snow density have been observed in the High Mountain Asia, however, its spatiotemporal patterns and underlying drivers remain incompletely understood. By integrating ERA5 and ERA5-Land reanalysis datasets with large-scale atmospheric circulation data, combined with advanced statistical methods, this study systematically analyzes the spatiotemporal patterns and driving factors of snow density across multiple scales in the High Mountain Asia. The results indicate that: The snow density exhibited a significant decreasing trend at a rate of −0.4 kg/m^3^·per decade (p < 0.01) from 1960 to 2023. Spatially, snow density consistently demonstrated a “high in mountains, low in plateaus” distribution pattern, which is closely associated with snow depth and snow accumulation. Significant decreases in snow density were concentrated in areas with relatively low snow accumulation, such as the southwestern (S_2_) and southeastern (S_3_) Tibetan Plateau, where snowpack exhibits higher sensitivity to temperature variations. Snow depth and air temperature serves as key geographical factor influencing snow density, the latter primarily affects snow density by modulating the proportion of snowfall in total precipitation and altering snow phenology. The East Atlantic/Western Russia (EA/WR) teleconnection pattern indirectly influences snow density through its control on temperature. A weakened EA/WR pattern facilitates increased advection of warm air from the southeast into the Asian High Mountain region, thereby elevating summer temperatures and contributing to reduced snow density.

## Introduction

The cryosphere is one of the five major components of the Earth’s climate system and plays a crucial role in global climate dynamics [1,2]. It not only influences the formation and development of climate but also exerts a significant feedback effect on global climate change. Snow cover, characterized by its high albedo, low thermal conductivity, and heat absorption during the melting process [3], represents the most extensive component of the cryosphere. It is both the most sensitive environmental change factor within the cryosphere [4], and the most active driver of environmental changes [5]. Snow density, defined as the mass of snow per unit volume, is a crucial parameter for estimating snow water equivalent, investigating watershed water balance, and simulating snowmelt runoff [6,7]. Therefore, in-depth exploration of the spatiotemporal variation of snow density and its driving mechanism is of great significance for predicting snowmelt runoff and evaluating the regional hydrological and water resources conditions.

Numerous scholars have conducted investigations into the changes in snow cover and their driving mechanisms. Regarding snowfall, Sun et al. estimated the mean and extreme snowfall in the Asian High Mountain region over the past 40 years using the MSWX dataset [8], while Li et al. assessed projected changes in snowfall under different warming scenarios based on NEX-GDDP high-resolution data [9]. In terms of snow cover area, several researchers have focused on specific regions of China, including northern Xinjiang, Northeast China, and the Tibetan Plateau [10–12], to explore the spatiotemporal characteristics and potential driving factors of snow cover extent. Concerning snow phenology, Zhao et al. and Tang et al. analyzed variations in the snow cover start (SCS), snow cover melt (SCM), and snow cover days (SCD) across China and the High Mountain Asia, respectively, identifying key geographical factors influencing these metrics [12,13]. As for snow density, Feng and Zhao investigated the spatiotemporal variation characteristics of snow density in the central Tianshan Mountains and the Tibetan Plateau, respectively [14,15]. Other studies have utilized in-situ observations to examine snow density variations across broader domains such as the Arctic and the Northern Hemisphere [15,16], however, these largely omitted mechanistic analyses of the observed changes. Overall, while considerable attention has been directed toward snow cover area, phenology, and snowfall variability, research on changes in the physical properties of snowpack, including snow density, remains relatively limited.

The High Mountain Asia is the area with the largest snow cover apart from the Arctic and Antarctic [3,17,18], and its snow cover fluctuations can serve as an indicator of regional and global climate change. Furthermore, the High Mountain Asia is also the source of many rivers including the Yangtze River, the Yellow River, the Mekong River, and the Yarlung Zangbo River [8], providing essential water resources for nearly 2 billion people in the surrounding areas [19]. Therefore, understanding the spatiotemporal variations of snow density in the High Mountain Asia and its influencing factors is of significant reference value for analyzing climate change and promoting sustainable economic development in the surrounding regions.

The research objectives of this paper mainly include: (1) revealing the variation characteristics of snow density in the High Mountain Asia at different temporal and spatial scales; (2) exploring the driving mechanisms of geographical factors (such as air temperature, precipitation, snow depth, etc.) and atmospheric circulation factors on snow density changes. The research results can enhance the understanding of snow cover changes in the High Mountain Asia and provide scientific references for regional water resource management.

### Study area overview

The High Mountain Asia (27°N ~ 45°N, 67°E ~ 104°E) refers to the high-altitude area in Asia centered on the Tibetan Plateau [3]. Covering an area of approximately 2.9 × 10^6^ km^2^, this region encompasses major mountain ranges and plateaus such as the Tianshan, Hindu Kush, Himalayas, Karakoram, and the Pamir Plateau [20], with an average elevation exceeding 4000 meters [21,22]. It contains about 100,000 km^2^ of glacier cover and a snow storage volume of approximately 163 km^3^ [23,24], making it the most ice- and snow-rich stable region outside the polar areas [25]. Owing to its extensive cryospheric storage and the vital contribution of glacier and snow meltwater to many major Asian rivers, the area is widely recognized as the “Asian Water Tower” [26,27].

The High Mountain Asia features a plateau mountain climate, characterized by predominant cold and arid conditions. The annual precipitation in these areas is approximately 400 mm, with more than 40% of it falling as snow [28]. The circulation patterns which influence and control the climate of the High Mountain Asia are relatively complex. The mid-latitude westerlies dominate areas including the Tianshan and the Hindu Kush Mountains, supplying substantial winter snowfall [29]. The southwestern Tibetan Plateau is primarily influenced by the South Asian monsoon, which contributes to enhanced summer snowfall [30,31].In the southeastern Tibetan Plateau, the East Asian and the South Asian monsoon exerts a strong influence [32–34].In order to study the spatial heterogeneity of snow density in this high-altitude zone, we have divided the High Mountain Asia into four sub-regions: the Tianshan-Hindu Kush Mountains (S_1_),the southwestern Tibetan Plateau (S_2_), the southeastern Tibetan Plateau (S_3_), and the northern Tibetan Plateau (S_4_) [35].

## Materials and methods

### Data sources and processing

The data of snow density, air temperature at 2m height, precipitation, snow evaporation, and snow depth are sourced from the ERA5-Land reanalysis dataset; the wind aspect and speed, temperature at 500 hPa pressure level are sourced from the ERA5 reanalysis dataset, both of which are provided by the ECMWF (https://cds.climate.copernicus.eu/). The ERA5 dataset covers the period from 1940 to the present at a horizontal resolution of up to 0.25° × 0.25°, while the ERA5-Land dataset is derived from the land component of the ERA5 via resampling, spans from 1950 to the present at an improved horizontal resolution of 0.1° × 0.1°.

In accordance with ECMWF definition, downward moisture flux is defined as positive. Therefore, to align with common interpretation, snow evaporation values were converted to their absolute values. Furthermore, the snow evaporation and precipitation in ERA5-Land dataset are daily averages, therefore, these two elements are multiplied by the number of days in each month respectively, thereby obtaining the total amount for each month. Following a quality assessment of the datasets, missing values were filled using the nearest-neighbor interpolation method. Subsequently, data for the relevant study period were extracted according to the specified temporal scope of the analysis.

The large-scale climate factor data used in the present research are: the Pacific North American Index (PNA), the Western Pacific Index (WP), the Eastern Atlantic/Western Russia Teleconnection Pattern Index (EA/WR), the Arctic Oscillation Index (AO), the North Atlantic Oscillation (NAO), the Southern Oscillation Index (SOI), the Eastern Tropical Pacific SST (Niño3), the East Central Tropical Pacific SST (Niño3.4), the Western Hemisphere Warm Pool (WHWP), the Oceanic Niño Index (ONI), the Northern Oscillation Index (NOI), the North Pacific Pattern (NP), the Trans-Niño Index (TNI), the Tropical Northern Atlantic Index (TNA). All these data were sourced from the NOAA website (https://psl.noaa.gov/data/climateindices/list/). These patterns represent important modes of internal variability in the Northern Hemisphere’s atmosphere and oceans, and may have a certain impact on the climate of the study area [37–42]. For instance, when the East Atlantic/West Russia (EA/WR) teleconnection pattern is in its positive phase during spring, an anticyclonic circulation anomaly tends to form south of Lake Baikal, which weakens the cold air masses affecting northwestern China [41]. Additionally, the Arctic Oscillation (AO) is the dominant mode of atmospheric circulation variability in the extratropical Northern Hemisphere. During its negative phase, the level pressure over mid-latitude high-pressure systems is lower than normal, facilitating southward intrusions of cold air [42].

**Fig 1:**
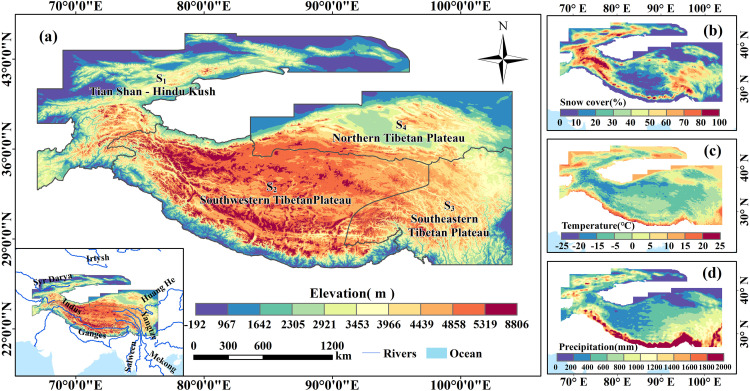
Overview of the study area. **(a)** The sub-regions and elevation of the High Mountain Asia, sub-region S_1_, S_2_, S_3_, S_4_ separately represents the Tianshan-Hindu Kush Mountains, the southwestern Tibetan Plateau, the southeastern Tibetan Plateau, the northern Tibetan Plateau. **(b–d)** Multi-year snow cover rate (unit: %), temperature (unit: °C), precipitation (unit: mm) in the study area, the data of three maps were obtained at ERA5-Land reanalysis dataset (https://cds.climate.copernicus.eu/). The HMA boundary used in Fig 1 is the result of the Randolph Glacier Inventory v6.0 [36], and it was modified with ArcGIS 10.8. The 50m world coastline used in Fig 1 was obtained at the following website: https://www.naturalearthdata.com/features.

The STRM elevation data used in this study comes from the “Geospatial Data Cloud” (http://www.gscloud.cn) with a horizontal resolution of 90 meters, and the geo-graphic coordinate system is GCS-WGS-1984. After stitching all the elevation images together, we extracted the elevation of the study area using the boundary of the High Mountain Asia, as illustrated in Fig 1.

### Methods

#### Correlational analysis.

The correlation coefficient was employed to examine the relationships between snow density and geographical factors, including air temperature, precipitation, snow evaporation, and snow depth, with statistical significance of the coefficients being assessed.

#### Mann-Kendall test.

The M-K test is a non-parametric statistical test approach, the main advantage of which is that it does not require a trend to be linear or data to follow a positive distribution, so it is widely applied in time series analysis in the fields of meteorology and climate change [43–45]. This method was applied to determine whether the trends in snow density were statistically significant.

#### Multiple stepwise regression analysis.

The Multiple Stepwise Regression method was used to establish the relationship between snow density in the High Mountain Asia and potential geographical factors in this study. The method progressively evaluates the significance of potential predictors, retaining those that contribute substantially to the variance in snow density. Based on the magnitude of variance explanation, and under the dual criteria of statistical significance (p < 0.05) for included variables and a minimum model explanatory power of 65%, the procedure identified the dominant geographical factors exerting significant influence on snow density.

## Results and analysis

### Characteristics of snow density changes at different spatial-temporal scales

#### Annual scale.

**Fig 2:**
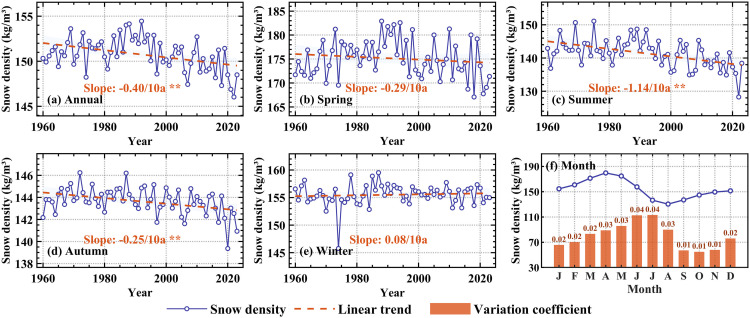
Variation of snow density in the High Mountain Asia at annual, seasonal scales. **(a–e)** Snow density trend of annual, spring, summer, autumn, winter in the High Mountain Asia from 1960 to 2023, * indicates that the 95% significance test is passed, and ** indicates that 99% significance test is passed. **(f)** The average monthly snow density in the High Mountain Asia and its coefficient of variation.

The annual average snow density in the High Mountain Asia exhibited a statistically significant decreasing trend (p < 0.01) from 1960 to 2023, at a linear rate of −0.4 kg/m^3^ per decade (Fig 2a). Prior to 1998, snow density remained relatively stable, with a multi-year average of 151.53 kg/m^3^. After 1998, however, it showed a fluctuating downward trend, with a multi-year average of 149.59 kg/m^3^.

**Fig 3:**
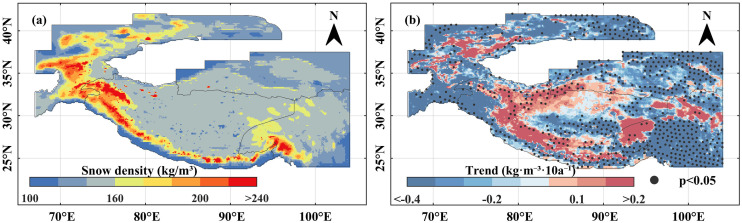
The spatial distribution  and variation characteristics of snow density in the High Mountain Asia from 1960 to 2023. **(a)** The spatial distribution of snow density in the High Mountain Asia. Snow density ranging from 100 to 160 kg/m^3^, 160–200 kg/m^3^ are considered as low value, medium value zone, while the snow density greater than 200 kg/m^3^ is classified as high value zone. **(b)** The spatial trend distribution of annual snow density in the High Mountain Asia. The black dots in the map indicate that the 95% significance test is passed. The High Mountain Asia boundary used in Fig 3 is the result of the Randolph Glacier Inventory v6.0 [36], and it was modified with ArcGIS 10.8.

Spatially, snow density across the High Mountain Asia during 1960–2023 was heterogeneous, demonstrating a general pattern of “higher in mountains, lower in plateaus”. Notably, high snow density values were predominantly clustered along a prominent arc-shaped mountain zone in Central Asia, extending from the Tianshan and Hissar ranges through the Alay, Hindu Kush, Karakoram, and Himalayan Mountains to the Hengduan Mountains (Fig 3a). The identified pattern of “high in mountains, low in plateaus” is primarily attributed to differences in temperature and snow depth. Although both topographic types belong to the broad category of mountainous terrain, significant differences exist in their mean elevations, with mountains generally attaining higher altitudes than plateaus. This elevational contrast leads to pronounced temperature disparities between the two landforms. The lower air and surface temperatures characteristic of high mountain areas facilitate prolonged snow persistence and greater accumulation depths. These conditions enhance processes such as snow compaction, thermal insulation, and hydrological effects, resulting in higher snow density in mountain regions.

In terms of spatial coverage, areas with low snow density (<160 kg/m^3^) accounted for the largest proportion of the study region. The central of southwestern Tibetan Plateau, the northeastern and southeastern Tibetan Plateau fell within this low-density category. By contrast, medium-to-high density regions (≥160 kg/m^3^) occupied only 21.9% of the study area and were predominantly concentrated in the Central Asian arc-shaped. Regarding spatial trends, snow density across the High Mountain Asia generally exhibited a decreasing trend, although some regions experienced increases (Fig 3b). Significantly decreasing regions were mainly distributed over the northeastern and southeastern Tibetan Plateau and the eastern Tianshan Mountains, accounting for 17.9% of the study area. In comparison, significantly increasing trends were observed in only 1.86% of the study area, primarily along the northern sectors of the Himalayas Mountains and the northwestern margin of southwestern Tibetan Plateau (S_2_).

#### Seasonal scale.

At the seasonal scale, snow density in the High Mountain Asia exhibited distinct seasonal variations. Spring and winter average values of 175.14 kg/m^3^ and 155.51 kg/m^3^, respectively, were higher than those in autumn (143.65 kg/m^3^) and summer (141.44 kg/m^3^). From 1960 to 2023, significant decreasing trends (p < 0.01) were observed in summer and autumn, with linear rates of −1.14 kg/m^3^ per decade and −0.25 kg/m^3^ per decade, respectively. In contrast, snow density in spring and winter remained relatively stable (Fig 2b–e).

**Fig 4:**
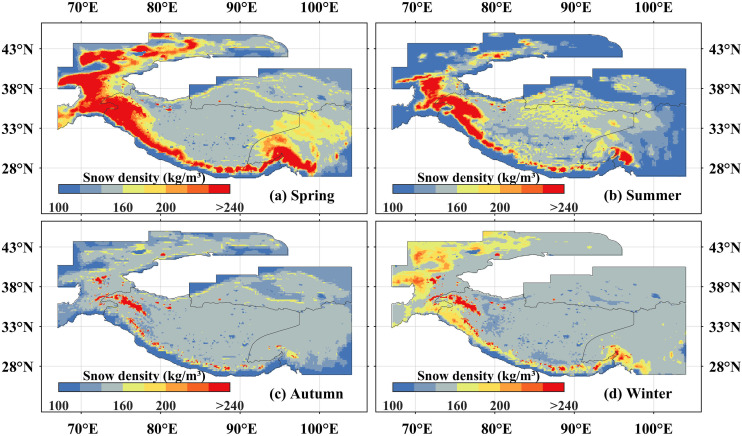
The spatial distribution of snow density in the high Mountain Asia at seasonal scale from 1960 to 2023. **(a–d)** The spatial distribution of snow density in the High Mountain Asia from spring to winter. **(a**–**d)** represent spring, summer, autumn, and winter, respectively. The High Mountain Asia boundary used in Fig 4 is the result of the Randolph Glacier Inventory v6.0 [36], and it was modified with ArcGIS 10.8.

Spatially, snow density across the High Mountain Asia demonstrated a consistent “high in mountains, low in plateaus” distribution pattern across all seasons (Fig 4). In each season, areas of high snow density remained concentrated along the Central Asian arc-shaped mountain zone, although the spatial extent of these medium-to-high density zones varied seasonally. Specifically, spring and winter displayed a larger coverage of medium-high snow density zones within the Central Asian arc-shaped mountain zone, whereas summer and autumn showed a reduced extent. This seasonal variation is primarily attributed to lower temperatures in winter and spring, which result in greater snow accumulation and snow depth in terms of both total quantity and depth within the zone. As a consequence, the effects of snow compaction, thermal insulation, and associated hydrological processes are more pronounced during these colder seasons. Furthermore, regions such as the Tian Shan, Qilian, and the Hengduan Mountains demonstrated a distinct “low in summer and autumn, high in winter and spring” pattern in snow density. Owing to their relatively lower overall elevation, snow accumulated during winter undergoes substantial melt in spring. By summer and autumn, seasonal snow cover persists only in limited high-altitude areas, resulting in reduced snow density across most of these regions.

**Fig 5:**
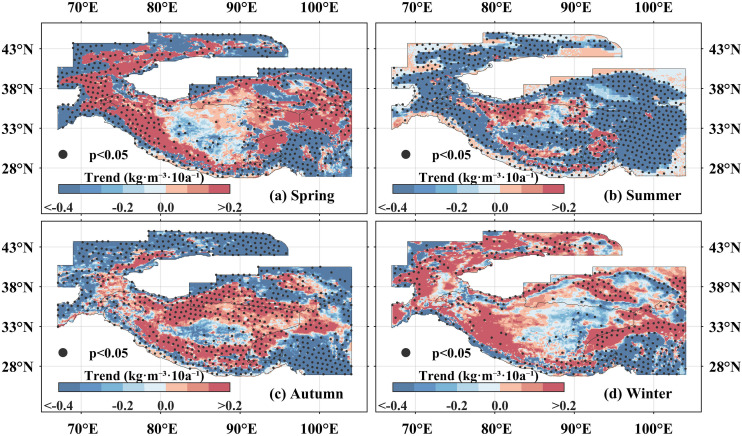
The spatial trend distribution of snow density in the High Mountain Asia at seasonal scale from 1960 to 2023. **(a–d)** Represent spring, summer, autumn, and winter, respectively. The blue series indicates a downward trend in snow density, while the red series represents an upward trend in snow density. The black dots in the map indicate that the 95% significance test is passed. The High Mountain Asia boundary used in Fig 5 is the result of the Randolph Glacier Inventory v6.0 [36], and it was modified with ArcGIS 10.8.

Regarding spatial trends, in spring, significantly decreasing trends covered 13.24% of the area, primarily concentrated in the Qilian Mountains, Hengduan Mountains, Tianshan Mountains, and the western Hindu Kush Mountains. Regarding spatial trends, in spring, significantly decreasing trends(p < 0.05) covered 13.24% of the High Mountain Asia, primarily concentrated in the Qilian Mountains, Hengduan Mountains, Tian Shan Mountains, and the western Hindu Kush (Fig 5a). In contrast, a significantly increasing trend was clustered in the western Himalayas, Pamir Plateau, and the Karakoram Mountains. During summer, areas exhibiting significant decreases in snow density were widespread, accounting for 39.60% of the High Mountain Asia (Fig 5b). The spatial distribution of significant decreases in autumn resembled that of spring, while the southern and northern Tibetan Plateau emerged as areas with significant increases (Fig 5c). In winter, the regions where snow density decreased significantly were localized in the Hengduan Mountains, whereas increase predominated in the southeastern Tibetan Plateau, Qaidam Basin, and eastern Tianshan Mountains (Fig 5d).

#### Monthly scale.

**Fig 6:**
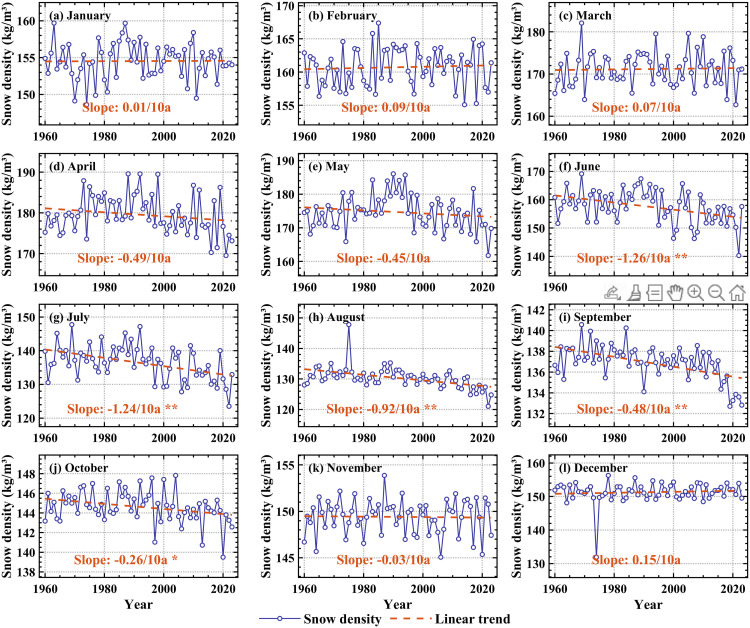
Variation of snow density in the High Mountain Asia at monthly scale from 1960 to 2023. **(a–l)** Represent January, February, March, April, May, June, July, August, September, October, November, and December respectively. * indicates that the 95% significance test is passed, and ** indicates that 99% significance test is passed.

As illustrated in Fig 6, the snow density in the High Mountain Asia experienced significant (p < 0.05) declining trends from June to October during 1960−2023, with the most pronounced rate of −1.26 kg/m^3^ per decade observed in these months. In the remaining months, snow density remained relatively stable. Fig 2f depicts the monthly mean snow density and its variation coefficients over the study period. Monthly snow density followed a unimodal distribution throughout the year, peaking in April and reaching its lowest value in August. Notably, the variation coefficient remained below 0.05 across all months, indicating low interannual variability in snow density at the monthly scale.

**Fig 7:**
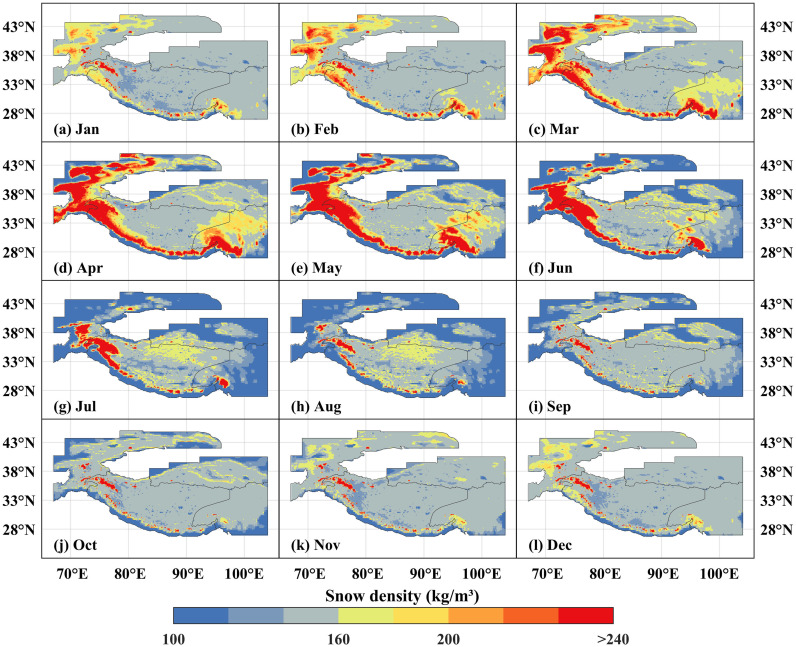
The spatial distribution of snow density in the high Mountain Asia at monthly scale from 1960 to 2023. **(a–l)** Correspond respectively to January, February, March, April, May, June, July, August, September, October, November and December. The High Mountain Asia boundary used in Fig 7 is the result of the Randolph Glacier Inventory v6.0 [36], and it was modified with ArcGIS 10.8.

Spatially, The High Mountain Asia showed a consistent “high in mountains, low in plateaus” spatial pattern persisted across all months, with pronounced spatial heterogeneity mainly observed across the Central Asian arc-shaped mountain zone and regions such as the Tianshan, Qilian, and Hengduan Mountains (Fig 7), other areas showed relatively stable snow density conditions. The spatial extent of medium-high snow density zones (≥160 kg/m^3^) within the Central Asian arc-shaped mountain zone showed distinct dynamics: gradual monthly expansion from January to April, progressive contraction from May onward, stabilization after August. From May to September, snow density in the Tianshan, Qilian, and Hengduan Mountains was notably lower than in other months, which can be primarily attributed to higher temperatures during this period, leading to reduced snow accumulation and shallower snow depth.

**Fig 8:**
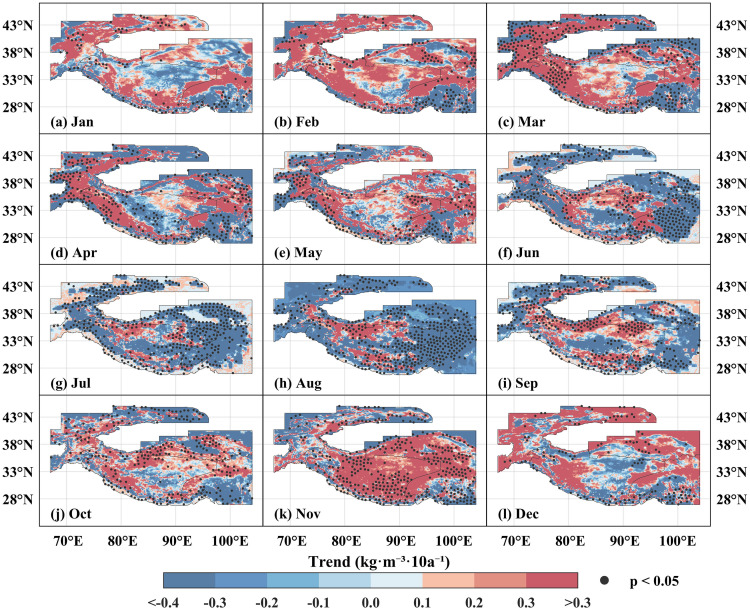
The spatial trend distribution of snow density in the High Mountain Asia at monthly scale from 1960 to 2023. **(a–l)** correspond respectively to January, February, March, April, May, June, July, August, September, October, November and December. The black dots in the map indicate that the 95% significance test is passed. The High Mountain Asia boundary used in Fig 8 is the result of the Randolph Glacier Inventory v6.0 [36], and it was modified with ArcGIS 10.8.

Regarding spatial trends, the snow density across the High Mountain Asia remained relatively stable from January to May over the past sixty-four years, with statistically significant increasing or decreasing regions accounting for less than 9.7% of the study area (Fig 8). In March, significant decreases in snow density were localized in the Hengduan Mountains and the northeastern sector of the Qilian Mountains, while significant increases in snow density were distributed in the western Tianshan Mountain, the Pamir Plateau, the Hindu Kush, and the western Himalayas Mountains. From June to August, the snow density in the study area generally decreased, with significant decrease concentrated in the Tianshan Mountains, the southeastern and northeastern parts of the Tibetan Plateau, and the Hengduan Mountains. During September to December, regions showing significant decreases in snow density were relatively scattered and occupied a low spatial proportion, whereas significant increases were mainly distributed along the southern and northern sectors of the Tibetan Plateau.

### Factors affecting snow density in the High Mountain Asia

#### Relationship between geographical factors and snow density.

To comparatively analyze the influencing factors of snow density variations across different regions, the High Mountain Asia (S) was divided into four sub-regions (Fig 1): the Tianshan-Hindu Kush Mountains (S_1_), the southwestern Tibetan Plateau (S_2_), the southeastern Tibetan Plateau (S_3_), and the northern Tibetan Plateau (S_4_).

Table 1 illustrates the Pearson correlation coefficients between snow density and four geographical factors in the High Mountain Asia. Snow density showed strong correlations with snow depth and air temperature. Throughout the entire study area and within individual subregions, snow density increased with greater snow depth and lower air temperature. In the Tianshan-Hindu Kush Mountains (S_1_), snow density was not only strongly related to snow depth and air temperature, but also exhibited a notable positive correlation with precipitation. In the southwestern (S_2_) and northern Tibetan Plateau (S_4_), snow evaporation also served as a significant factor affecting snow density, with increased snow evaporation corresponding to higher snow density.

**Table 1:** Correlation coefficients between snow density and geographical factors in the High Mountain Asia.

	Region	Temperature (°C)	Precipitation (mm/a)	Snow evaporation (mm/a)	Snow depth (mm)
Coefficient	S	−0.39**	0.35**	0.36**	0.89**
	S_1_	−0.33**	0.51**	0.29**	0.90**
	S_2_	−0.32**	0.13	0.43**	0.86**
	S_3_	−0.70**	0.29	0.28	0.93**
	S_4_	−0.67**	0.11	0.79**	0.69**

** Indicate that the correlation was significant at the 99% confidence level.

To identify the dominant geographical factors influencing snow density in different regions, stepwise regression models were constructed separately. The coefficients of determination (R^2^) for these models ranged from 0.68 to 0.88, indicating a satisfactory model performance on identifying the dominant factor of snow density.

The regression results revealed that snow depth was the primary controlling factor of snow density in the High Mountain Asia, as well as in the Tianshan-Hindu Kush Mountains (S_1_), southwestern Tibetan Plateau (S_2_), and southeastern Tibetan Plateau (S_3_) subregions. In the northern Tibetan Plateau (S_4_), however, snow evaporation becomes the main factor influencing snow density.

#### Relationship between circulation factors and snow density.

**Fig 9:**
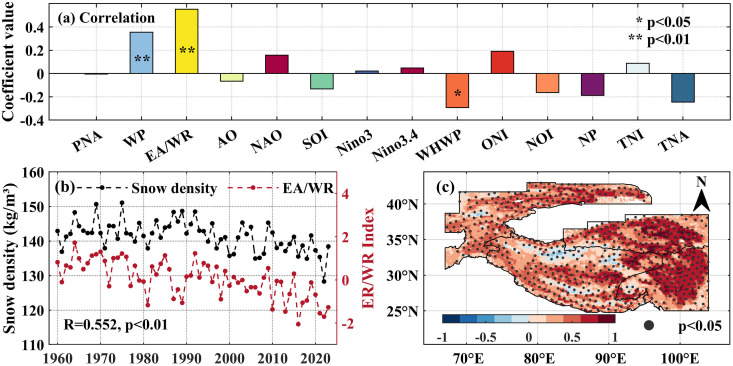
The relationship between summer snow density in the high Mountain Asia and the corresponding circulation factors. (a) The bar chart shows the correlation coefficient between summer snow density in the High Mountain Asia and the corresponding circulation factors, * and ** indicate that the 95%, 99% significance test is passed, respectively. **(b)** Time series of summer snow density (black line segment) High Mountain Asia and EA/WR teleconnection pattern index (red line segment). **(c)** Spatial correlation distribution between summer snow density and EA/WR teleconnection pattern index, the black dots in the map indicate that the 95% significance test is passed. The High Mountain Asia boundary used in Fig 9 is the result of the Randolph Glacier Inventory v6.0 [36], and it was modified with ArcGIS 10.8.

Given the most rapid decline in snow density during summer, this study focuses on diagnosing the circulation drivers behind summer snow density variability in the High Mountain Asia. Fig 9a illustrates the correlation coefficients between summer snow density in the High Mountain Asia and 14 large-scale circulation factors. The strongest correlation was identified with the East Atlantic/West Russia (EA/WR) teleconnection pattern (R = 0.552, p < 0.01), such that a decline in the EA/WR index corresponded to lower snow density. Spatially, the summer snow density was positively correlated with the EA/WR teleconnection pattern index at most grid points, with the spatial proportion of significantly positive correlation grid points reaching 42.96% (Fig 9c).

**Fig 10:**
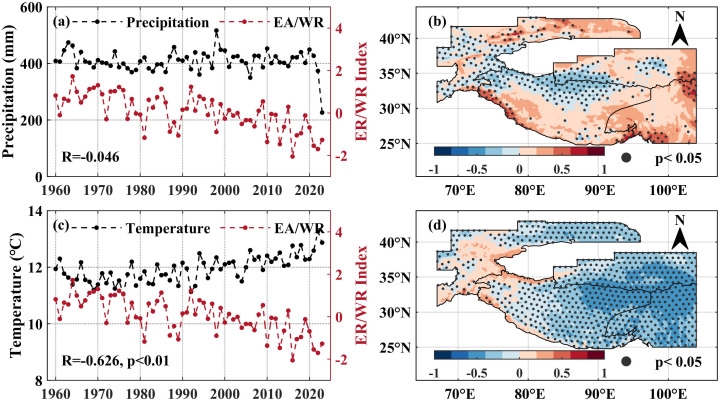
The spatiotemporal correlation between summer precipitation, temperature in the High Mountain Asia and EA/WR teleconnection pattern. **(a)** Time series of summer precipitation (black line segment) in the High Mountain Asia and EA/WR teleconnection pattern index (red line segment). **(b)** Spatial correlation distribution between summer precipitation and EA/WR teleconnection pattern index, the black dots in the map indicate that the 95% significance test is passed. **(c)** Time series of summer temperature (black line segment) in the High Mountain Asia and EA/WR teleconnection pattern index (red line segment). **(d)** Spatial correlation distribution between summer temperature and EA/WR teleconnection pattern index, the black dots in the map indicate that the 95% significance test is passed. The High Mountain Asia boundary used in Fig 10 is the result of the Randolph Glacier Inventory v6.0 [36], and it was modified with ArcGIS 10.8.

The influence of circulation factors on snow density is not direct; but is typically mediated through their control over temperature and precipitation, which in turn affect snowpack properties. To verify whether the East Atlantic/West Russia (EA/WR) teleconnection pattern correlates with summer precipitation and temperature in the High Mountain Asia, this study calculated the overall and pixel-wise correlation coefficients between the summer EA/WR teleconnection pattern index and the contemporaneous precipitation, temperature across the study area. At the regional scale (Fig 10a, 10c), the EA/WR index showed correlation coefficients of −0.046 with precipitation and −0.626 (p < 0.01) with temperature, indicating a stronger influence on temperature but a weak association with precipitation.

Spatially, significant negative correlations (p < 0.05) between precipitation and the EA/WR index were concentrated on the northern sector of southwestern Tibetan Plateau, the Pamir Plateau, and the Karakoram Mountains (Fig 10b), covering 23.17% of the study area, with a mean correlation coefficient of −0.367. The spatial pixels with a significant positive correlation with precipitation are less than 5%. In contrast, temperature showed significant negative correlations with the EA/WR index across most pixels, accounting for 78.39% of the spatial area (Fig 10d), with a mean correlation coefficient of −0.515.

## Discussion

### The mechanism of geographic factors on snow density

Snow depth serves as a key geographic factor driving snow density variations in the High Mountain Asia. The underlying mechanisms include: (1) Greater snow depth enhances compaction under gravitational forcing, promoting snow densification [46]. (2) Deeper snow depth improved thermal insulation of the snowpack, protecting it from external climatic fluctuations [46,47]. (3) Due to the hydrological effects within the snowpack, increased snow depth facilitates the meltwater percolation and refreezing processes, thereby filling pore spaces and increasing density [48]. (4) Enhanced snow depth mitigates ablation and evaporative losses under warming conditions by increasing the energy required for melt, thereby slowing the rate of snow density reduction.

Air temperature also plays a critical role in regulating snow density across the study area. On one hand, temperature influences the proportion of solid precipitation [19,25,49], thereby affecting total snowfall accumulation and snow depth [13,50]. On the other hand, rising temperatures alter snow phenology-delaying the first snow date (SCS), advancing the melt (SCM), and shortening the snow cover days (SCD), which collectively reduce snow accumulation and decrease snow depth [13,25]. Notably, the influence of air temperature on snow density is more pronounced in the southeastern (S_3_) and northern (S_4_) Tibetan Plateau compared to other subregions. This heightened sensitivity can be attributed to two main factors:(1) The generally shallower snowpack in these areas is more vulnerable to external thermal influences, particularly under sustained warming; (2) The snow climate in the southeastern and northern Tibetan Plateau is more strongly affected by warm, moist air masses from the east, whereas the southwestern plateau and Tianshan regions are predominantly influenced by the westerly circulation patterns.

### The mechanism of circulation factors on snow density

**Fig 11:**
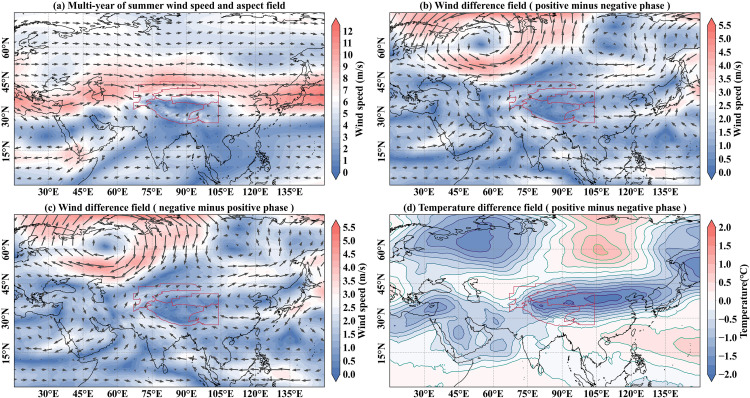
Circulation mechanism of summer snow density in the High Mountain Asia. **(a)** The multi-year of summer wind speed and aspect field in the Northern Hemisphere. The direction of the arrow represents the wind direction, and the length of the arrow indicates the wind speed. **(b)** The 500 hPa wind difference field of EA/WR teleconnection pattern (typical positive phase minus negative phase). **(c)** The 500 hPa wind difference field of EA/WR teleconnection pattern (typical negative phase minus positive phase). **(d)** The temperature difference field of EA/WR teleconnection pattern (typical positive phase minus negative phase). The High Mountain Asia boundary used in Fig 11 is the result of the Randolph Glacier Inventory v6.0 [36], and it was modified with ArcGIS 10.8. The 50m world coastline used in Fig 11 was obtained at the following website: https://www.naturalearthdata.com/features.

Fig 11a illustrates the average summer field of 500 hPa wind speed and aspect over the Northern Hemisphere from 1960 to 2023. Westerly flows prevail between 20°N and 75°N, which are obstructed and deflected by the Tibetan Plateau and other major topography. This results in the splitting of the westerlies into northern and southern branches around the plateau, while a weak wind zone forms over the central Tibetan Plateau due to blocking by its western mountainous margins.

To understand the circulation patterns in the High Mountain Asia during typical positive and negative phase years, representative years of EA/WR teleconnection pattern positive and negative phases were selected to synthesize the 500 hPa wind field and temperature field in the study area, thereby analyzing the impact of EA/WR teleconnection pattern on snow density. Fig 11b and Fig 11c illustrate the difference in circulation fields between positive and negative phases of EA/WR teleconnection pattern. The wind difference field shows easterly or northeasterly anomalies over the High Mountain Asia during positive EA/WR phases, indicating increased incursion of dry, cold air from higher latitudes. This transport leads to lower temperatures over the High Mountain Asia, which is conducive to an increase in snow density. In contrast, negative EA/WR phases are associated with enhanced warm, moist air advection from lower-latitude oceans, raising temperature of the study area and promoting lower snow density.

The summer 500 hPa temperature difference field across the Northern Hemisphere (Fig 11d) reveals a prominent cold center over the High Mountain Asia during positive EA/WR phases, further confirming cooler summer temperatures in the study area under such conditions. This thermal regime supports enhanced snow accumulation and persistence, correspondingly contributing to increased snow density.

This study has two primary limitations that warrant attention in future research. Although the key relationships among snow density, meteorological factors, and snow depth have been elucidated, the analysis did not account for terrain variables such as elevation, slope, and aspect. These topographic parameters significantly influence the spatial redistribution of temperature and precipitation. Subsequent investigations should aim to clarify the mechanisms through which terrain modulates snow physical properties. Additionally, while several circulation factors potentially affecting snow density in the High Mountain Asia were investigated, other large-scale atmospheric processes may also exert considerable influence. Further in-depth studies are required to explore these additional circulation drivers and their interactions with regional snowpack characteristics.

## Conclusions

This study analyzed the spatiotemporal variation in snow density in the High Mountain Asia and revealed the underlying driving factors. Our main conclusions are summarized as follows:

Snow density exhibited a statistically significant decreasing trend (p < 0.01) at a rate of −0.4 kg/m^3^ per decade from 1960 to 2023. A pronounced decline occurred in summer and autumn (p < 0.01), with a rate of −1.14 kg/m^3^ per decade and −0.25 kg/m^3^ per decade, respectively, while the snow density remained relatively stable in winter and spring.

At multiple temporal scales, snow density consistently displayed “high in mountains, low in plateaus” spatial pattern. This distribution is closely linked to snow accumulation and snow depth, and fundamentally reflects temperature controls on snowpack properties.

Spatially, significant decreases in snow density were mainly observed in the Tianshan Mountains, the southwestern Tibetan Plateau (S_2_), and the southeastern Tibetan Plateau (S_3_). This phenomenon is largely attributed to the relatively less snow accumulation in these regions, which heightens their sensitivity to temperature changes.

Snow depth and air temperature are crucial geographical factors controlling snow density variability across the High Mountain Asia. Within a certain range, snow density increases with lower temperatures and greater snow depth. Moreover, the weakening of the East Atlantic/Western Russia (EA/WR) teleconnection pattern in summer enhances the advection of warm air from the southeast into the study area, raising summer temperatures and thereby contributing to reduced snow density.
